# Biogenesis and Dynamics of the Coronavirus Replicative Structures

**DOI:** 10.3390/v4113245

**Published:** 2012-11-21

**Authors:** Marne C. Hagemeijer, Peter J.M. Rottier, Cornelis A.M. de Haan

**Affiliations:** Virology Division, Department of Infectious Diseases & Immunology, Faculty of Veterinary Medicine, Utrecht University, Yalelaan 1, 3584 CL Utrecht, The Netherlands; Email: mch143@andromeda.rutgers.edu (M.C.H.); p.rottier@uu.nl (P.J.M.R.)

**Keywords:** coronavirus, replication complex, nonstructural protein, membrane rearrangements, protein-protein interactions, live cell imaging, dynamics, RNA synthesis

## Abstract

Coronaviruses are positive-strand RNA viruses that are important infectious agents of both animals and humans. A common feature among positive-strand RNA viruses is their assembly of replication-transcription complexes in association with cytoplasmic membranes. Upon infection, coronaviruses extensively rearrange cellular membranes into organelle-like replicative structures that consist of double-membrane vesicles and convoluted membranes to which the nonstructural proteins involved in RNA synthesis localize. Double-stranded RNA, presumably functioning as replicative intermediate during viral RNA synthesis, has been detected at the double-membrane vesicle interior. Recent studies have provided new insights into the assembly and functioning of the coronavirus replicative structures. This review will summarize the current knowledge on the biogenesis of the replicative structures, the membrane anchoring of the replication-transcription complexes, and the location of viral RNA synthesis, with particular focus on the dynamics of the coronavirus replicative structures and individual replication-associated proteins.

## 1. Introduction

Positive-strand RNA (+RNA) viruses are the most abundant viruses in nature. Many important pathogens belong to this category, including poliovirus (PV), hepatitis C virus (HCV) and the severe acute respiratory syndrome (SARS)-coronavirus (CoV). A distinctive common feature of +RNA viruses is the replication of their genomes in the cytoplasm of the host cell in association with rearranged cellular membranes that are remodeled into organelle-like membranous structures to which the viral replication-transcription complexes (RTCs) localize. 

The various membrane rearrangements observed in +RNA virus-infected cells range in size from 40 to 400 nm, contain lipids that are derived from various cellular compartments and demonstrate an impressively diverse plethora of morphologies that include, among others, clusters of vesicles for the *Picorna*- and *Togaviridae*, spherule-like invaginations for the *Bromoviridae* and *Nodaviridae*, and vesicle packets and membranous webs for the *Flaviviridae* (reviewed in [1,2,3]). These membrane rearrangements seem to be beneficial for (i) sequestering and concentrating all viral and cellular components necessary for viral RNA synthesis and (ii) to provide a protective microenvironment against virus-elicited host defense mechanisms. 

Also coronaviruses (CoVs), enveloped +RNA viruses that belong to the family *Coronaviridae*, extensively rearrange cellular membranes into organelle-like replicative structures during infection. These replicative structures consist of double membrane vesicles (DMVs) and convoluted membranes (CMs). The viral replicase proteins involved in RNA synthesis localize to both these structures, while double-stranded RNA (dsRNA), presumably functioning as replicative intermediate during viral RNA synthesis, has been detected at the DMV interior [4,5]. Recent studies have provided new insights into the assembly and functioning of the CoV replicative structures. This review will summarize the current knowledge on the biogenesis of the replicative structures, the membrane anchoring of the RTCs, and the location of viral RNA synthesis, with particular focus on the dynamics of the coronavirus replicative structures and individual replication-associated proteins.

## 2. Coronavirus Genome Organization and Gene Expression

The *Coronaviridae* are a family of evolutionary related, enveloped +RNA viruses that together with the *Arteriviridae* and the *Roniviridae*, belong to the order of the *Nidovirales*. Historically, CoVs have been recognized as important infectious agents for domestic livestock, poultry and companion animals. In contrast to the animal viruses, human CoVs (HCoVs) have been associated with relatively mild upper and lower respiratory tract infections, including ordinary common colds. However, in 2002–2003 the outbreak of a novel HCoV in China, causing severe fatal atypical pneumonia in infected individuals, demonstrated that HCoVs are also able to induce severe life-threatening disease in humans. This virus was called the SARS-CoV [6,7] and emerged in the human population from an animal reservoir, probably originating from bats, with palm civet cats acting as intermediate hosts [8,9]. 

**Figure 1:**
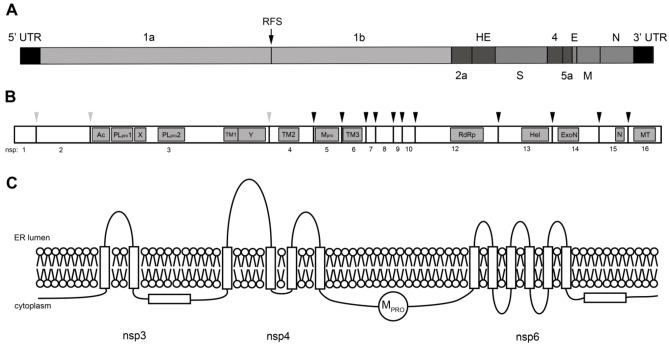
**Schematic representation of the coronavirus mouse hepatitis virus (MHV)-A59 genome, replicase polyprotein organization and membrane topology.** (**A**) Schematic representation of the +RNA genome of MHV-A59. The coronavirus genome contains a 5' cap structure and a 3' poly(A) tail, together with untranslated regions (UTRs). The first two-thirds of the genome consist of two large open-reading frames (ORFs), ORF1a and ORF1b, which are translated into two large replicase polyproteins (pp1a and pp1ab). Pp1ab is synthesized via a −1 ribosomal frameshift mechanism at the end of ORF1a (RFS). The final one-third of the genome contains the canonical CoV structural proteins-encoding genes (S, E, M and N), interspaced by several accessory genes (2a, HE, 4, 5a); (**B**) A schematic representation of pp1ab is shown. The coronavirus polyproteins are processed by viral proteinases residing in nsp3 (PLpro1 and PLpro2; grey arrowheads indicate cleavage sites) and nsp5 (Mpro; black arrowheads indicate cleavage sites), thereby generating 16 mature nsps. Hydrophobic domains (TM1, TM2 and TM3) in nsp3, nsp4 and nsp6 are indicated, together with predicted and identified RNA(-modifying) enzymes: the RNA-dependent RNA polymerase (RdRP; nsp12), the helicase (Hel; nsp13), the exonuclease (ExoN; nsp14), the uridylate-specific endoribonuclease (N; nsp15), and the methyl transferase (MT; nsp16); (**C**). Schematic representation of the topology of the coronavirus polyprotein. Only the part of the polyprotein is shown that contains the hydrophobic domains (indicated by boxes) residing in nsp3, nsp4 and nsp6. Nsp3 and nsp6 contain hydrophobic domains that do not span the lipid bilayer. Mpro indicates the viral protease residing in nsp5, in between nsp4 and nsp6.

Among the +RNA viruses, CoVs clearly distinguish themselves by carrying the most complex and largest genomes, which range in size from ~26 to 32 kb [10]. Despite the variation in size, the overall genome organization of the various CoVs is quite conserved. The genome contains all the genetic information necessary to direct both the synthesis of full-length genomic RNA (replication) and the (discontinuous) production of subgenomic mRNAs (transcription) [11]. The linear +RNA genome of CoVs is 3' polyadenylated and has a 5' cap structure, thereby mimicking cellular mRNAs. The 5' and 3' ends of the genome contain untranslated regions (UTRs) with *cis*-acting elements that are important for replication and transcription. Two-thirds of the genome consists of two large open reading frames (ORFs), ORF1a and ORF1b. The remaining 3' one-third part encodes the structural proteins interspersed with sequences encoding some accessory proteins. A schematic picture of the prototype mouse hepatitis virus (MHV) genome is shown in Figure 1A. 

The structural and the accessory proteins are expressed from a nested set of 3' coterminal subgenomic (sg) mRNAs that are generated via discontinuous transcription during subgenome-length minus-strand RNA synthesis [12,13]. The RNA-dependent RNA polymerase (RdRp) copies the genomic positive-sense RNA into a negative-sense template until it reaches a transcription-regulation sequence (TRS). At this point, RNA synthesis may either continue or the RdRp may relocate to the 5' end of the genome and complete the negative-sense sgRNA. These negative-sense sgRNAs serve as templates for the synthesis of the corresponding positive-sense sgRNAs. As a result, the positive-sense sgRNAs form a nested set of mRNAs, which extend for different lengths from a common 3' terminus while also having a common 5' end, which is known as the leader sequence. Generally, only the 5' first unique gene of each sgRNA is translated (reviewed in [11]). Minus- and plus-strand RNA synthesis is already detected at 75 to 90 min post infection (p.i.) [11]. Minus-strand synthesis, of which the resulting RNA species are mainly present as double-stranded intermediates because of their association with plus-strand RNA molecules, peaks at 5 to 6 h p.i. after which the synthesis declines but does not stop [14]. The plus-stranded RNAs are produced in a 50- to 100-fold excess over their minus-strand counterparts [11,15]. 

The viral replicase is encoded by the two most 5' ORFs of the genomic RNA, ORF1a and ORF1b. Translation of ORF1a and ORF1b of the genomic RNA generates two very large replicase polyproteins (pp), pp1a and pp1ab. The latter is synthesized via a −1 ribosomal frameshift mechanism mediated by a pseudoknot structural element at the end of ORF1a [16,17]. These replicase polyproteins are extensively processed by viral proteinases (reviewed in [18]), resulting in the generation of sixteen nonstructural proteins (nsps). A schematic representation of the CoV replicase polyprotein is shown in Figure 1B.

## 3. Coronavirus Nonstructural Proteins

### 3.1. Viral Proteinases

The CoV nsps form together with the nucleocapsid (N) protein, and presumably several host proteins, the membrane-associated RTCs. To generate the functional CoV replication complexes, the replicase polyproteins pp1a and pp1ab have to be proteolytically processed to liberate the sixteen individual protein products. Cleavage of pp1a and pp1ab is performed by two viral proteinases that reside in nsp3 and nsp5: the papain-like proteases (PLpro1 and PLpro2) located in nsp3 and the chymotrypsin-like cysteine proteinase (3CLpro), or main protease (Mpro), present in nsp5 (reviewed in [18]). Besides the mature nsps, also the intermediate and precursor polyproteins are likely to be functionally important. Hence, the cleavage of these proteins may somehow be involved in the temporal regulation of plus and/or minus sense viral RNA synthesis [18,19,20].

### 3.2. RNA-modifying Enzymes

The key enzyme involved in genome replication is the RNA-dependent RNA polymerase (RdRp), which is present in nsp12 [10]. The nsp12 protein is able to utilize both homo- and heteropolymeric RNAs as template but its RdRp activity is dependent on primers to copy the viral RNA [21]. These primers might be produced by a non-canonical RdRp activity that has been described for the nsp8-encoded ‘RNA primase’, as this protein is able to produce short oligonucleotides complementary to the RNA genome [22]. Nsp8 has been shown to associate together with nsp7 into a hexadecameric complex, consisting of eight copies of each protein, thereby forming a channel that can harbor RNA and may serve as a processivity factor for nsp12 [23]. Nsp8 also interacts with nsp7 in a 1:2 ratio [24] and is able to synthesize much longer transcripts [24,25].

The CoV nsp13 protein contains a superfamily 1 helicase domain with an amino-terminal zinc-binding domain that is important for the unwinding activity of duplex RNA (and DNA) in a 5'-to-3' direction [26,27,28]. The resulting single-strands probably serve as templates for RNA synthesis. The multifunctional nsp13 protein additionally possesses nucleotide triphosphatase activity [27,29,30] and is likely to be involved in removal of one of the terminal phosphate groups at the 5' end of the positive-sense RNAs, which is the first step in the formation of the 5' cap structure. Although the enzyme that subsequently adds the guanine to the terminal phosphates (guanylyl transferase) has not been identified yet, nsp14 has been shown to exert S-adenosyl-L-methionine (AdoMet)-dependent (guanine-N7)-methyltransferase (N7-MTase) activity [31]. Finally, the cap-1 structure is formed by the AdoMet-dependent (nucleoside-2'O)-methyltransferase (2'O-MTase) activity that is present in nsp16 [32], and for which the latter needs to form a complex with nsp10 [33,34]. 

CoVs possess the largest genomes among the +RNA viruses [10]. They encode a large number of additional RNA-modifying enzymes, which are often not present in other RNA viruses. These additional enzymatic activities are probably required to ensure proper RNA synthesis and might account for their large size. In addition to the cap N7-MTase activity, nsp14 has metal ion-dependent 3'-to-5' exoribonuclease (ExoN) activity [35] and contains a nidoviral uridylate-specific endoribonuclease (NendoU; nsp15) [36], both able to degrade RNA and dsRNA [36,37]. While the function of the NendoU activity in the CoV infection cycle is not known, it appears that the ExoN activity is required to ensure high replication fidelity of the extremely large CoV genome [38,39] and that nsp14 may function as a proofreading enzyme [40].

### 3.3. Nonstructural Transmembrane Proteins

CoVs encode three nsps that contain hydrophobic stretches that are predicted to function as transmembrane domains: nsp3, nsp4 and nsp6. Consistently, membrane association has been demonstrated for the nsp3, nsp4 and nsp6 proteins of SARS-CoV [41,42,43] and MHV [42,43,44,45]. Both nsp3 and nsp4 become *N*-glycosylated upon insertion into the membranes of the endoplasmic reticulum (ER) [42,43,44]. Interestingly, transmembrane domain predictions based on the multiple alignment of 27 CoV replicase polyprotein sequences revealed an uneven number of hydrophobic domains for both nsp3 and nsp6 [42]. This prediction is peculiar as it would separate the viral proteinases residing in nsp3 and nsp5 from their target sequences, implying that some of these hydrophobic domains might actually not span the membrane. In agreement herewith, and in contrast to the predictions, all three nsps were shown to have both their amino terminus and their carboxy terminus exposed in the cytoplasm. While all four hydrophobic domains of nsp4 span the lipid bilayer, this is the case for only two of the three hydrophobic domains in nsp3 and for six of the seven in nsp6 [42,45]. This experimentally established topology model (Figure 1C) makes more sense, as it positions all of the proteinase cleavage sites on the same side of the membrane as the viral proteinases themselves. Proteolytic processing of the replicase polyproteins probably starts during translation and prior to membrane insertion. Interestingly, the cleavage between nsp3 and nsp4 appears to be a rapid event [41,46,47]. This cleavage at the amino-terminus of the first hydrophobic/transmembrane domain of nsp4 probably facilitates the membrane insertion of nsp4 as it may enable the first hydrophobic domain to function as a signal peptide. The occurrence of conserved non-membrane spanning hydrophobic domains in nsp3 and nsp6, which are likely to be peripherally associated with the membrane, suggests an important function for these domains, possibly in the biogenesis of the CoV replicative structures. In this respect it is of interest to mention that the seventh hydrophobic domain of nsp6 contains putative palmitoylation sites (our own predictions and [45]). The addition of palmitic acid to this hydrophobic domain may stabilize its peripheral membrane association.

### 3.4. Nonstructural Proteins with Other Functions

In addition to the nsps mentioned above, other CoV nsps are involved in RNA binding (nsp9 and nsp10; [48,49]) or in evasion of the antiviral response of the host (nsp1 and nsp3; [50,51,52,53,54,55,56,57]). The function of nsp2 is not yet known, although this protein was shown not to be essential for virus replication [58,59]. The reader is referred to several excellent reviews on this topic for more detailed insights [10,60,61].

## 4. Membrane Rearrangements

### 4.1. Organelle-like Membranous Replicative Structures

**Figure 2:**
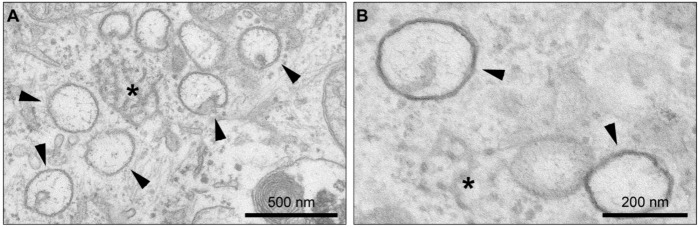
**Coronavirus-induced organelle-like replicative structures.** (**A**) Upon coronavirus infection, replicative structures consisting of double-membrane vesicles (DMVs) and convoluted membranes (CMs) are generated; (**B**) A higher magnification clearly demonstrates that the DMVs contain a double-lipid bilayer. The DMVs are indicated by arrowheads and the CMs by asterisks. The size of the scale bars is indicated.

Upon infection of host cells, CoVs induce a variety of membranous structures of which some have been associated with viral RNA synthesis. The first detectable membrane rearrangements in CoV-infected cells are 200 to 350 nm organelle-like structures that have been described for both MHV [47,62] and the SARS-CoV [5,63] and consist of spherical vesicles containing double lipid bilayers, termed DMVs (Figure 2). In between the clusters of DMVs, reticular CMs are characteristically present [4,62,64]. Later in infection large virion-containing vesicles (LVCVs) [4,5,62,65], highly organized cubic membrane structures [5,62] and condensed tubular bodies [62,64] are formed. The latter two structures are likely a result of the overexpression of CoV structural proteins during infection and do not seem to be involved in CoV replication [62].

Electron tomography studies demonstrated that in SARS-CoV infected cells the DMVs and CMs form an interconnected membranous network that is also continuous with the ER [4]. This latter observation is in agreement with previous reports describing DMVs in close proximity to the ER or continuous with it [5,47,63]. Moreover, (partial) colocalization of replicase proteins together with the ER resident protein disulfide isomerase (PDI) has been reported [63], while also the translocon subunit Sec61α was found to be redistributed to the replicative structures upon SARS-CoV infection [66]. The combined data indicate that the ER is the most likely membrane donor for the DMVs, despite the absence of most conventional ER markers on these structures [43,62,63,67].

The CoV replicative structures, *i.e.*, DMVs and CMs (Figure 2), have been associated with viral RNA synthesis, as the MHV and SARS-CoV nsps have been shown to localize to these structures [4,5,19,43,59,62,63,68,69,70,71]. In addition, antibodies recognizing dsRNA, the presumed replicative intermediates, label the interior of the SARS-CoV-induced DMVs [4]. Newly synthesized viral RNA, visualized by 5-bromouridine 5'-triphosphate (BrUTP) labeling, was observed in MHV-infected cells in close proximity to the replicative structures by immunoelectron microscopy [47,71].

### 4.2. Involvement of Cellular Pathways

Remodeling of eukaryotic cellular membranes into the replicative structures is likely dependent on the combined effects of both viral and cellular proteins and probably also on the specific lipid composition of the membranes themselves. Nevertheless, only few studies have been published addressing the involvement of cellular constituents in CoV replication and the generation of the replicative structures. It is conceivable that CoVs hijack cellular pathways to meet the conditions that are required for their replication, consequently adopting intrinsic properties of the utilized pathways themselves.

In agreement with the ER being the most likely membrane donor of the DMVs, an intimate association between the early secretory pathway and CoV replication has been demonstrated. RTC formation and replication in MHV-infected cells were inhibited when the secretory pathway was interfered with by blocking protein export at ER exit sites by treatment with the kinase inhibitor H89 or by expression of a dominant active Sar1 mutant [43]. Also treatment with Brefeldin A (BFA), an inhibitor of ER-to-Golgi trafficking, or knockdown of its target GBF1, inhibited MHV replication while reducing the number of DMVs [67]. Similar results were published for SARS-CoV infected cells treated with BFA and it was noticed that the inner and outer membranes of the DMVs were separated in BFA-treated cells [66], which may explain the observed inhibition of viral replication.

By their main ultrastructural characteristic, the double lipid bilayer, CoV DMVs very much resemble autophagosomes. This similarity prompted studies into the role of the autophagy machinery in CoV replication. Initial studies revealed a colocalization between the autophagosomal protein marker microtubule-associated protein light-chain 3 (LC3/Atg8) with the replicative structures [68]; moreover, viral replication was impaired and DMVs were not detected in the absence of the essential autophagy protein Atg5 [72]. In other studies however, these colocalization data could not be reproduced [63], while the absence of Atg5 did not affect CoV replication [73,74]. Yet, LC3 was found in association with the replicative structures by Zhao and coworkers [73]. Furthermore, others showed that, while the endogenous LC3 protein was recruited to the replicative structures, this was not the case for a GFP-tagged form of LC3 [69,75,76] that is often used as a marker for autophagosomes [77]. In agreement herewith, CoV replication was shown to be unaffected in autophagy-deficient cells lacking Atg7, although depletion of LC3 severely affected CoV replication [69]. Unlike autophagosomes, which may also be induced by expression of CoV nsp6 [74], CoV replicative structures were shown to be decorated with the non-lipidated form of LC3. Similar findings were also reported for EDEMosomes [78,79], ER-derived vesicles that transport ER chaperones to lysosomes. As the EDEMosome cargo proteins EDEM1 and OS-9 were also detected in association with the CoV replicative structures, it was proposed that CoVs hijack EDEMosomes for their replication [69,80]. In agreement herewith, the transmembrane SEL1L protein, which was recently shown to interact with LC3 and to have a critical function in EDEMosome biosynthesis, was also shown to colocalize with dsRNA foci in CoV-infected cells, while its depletion negatively affected CoV replication [80].

### 4.3. Role of Viral Proteins

The CoV nonstructural membrane proteins, nsp3, 4 and 6 probably play an essential role in the membrane rearrangements required for the induction of the replicative structures and in the anchoring of the RTCs to these structures. These proteins were shown to be engaged in homo- and heterotypic interactions [81]. Interestingly, co-expression of nsp4 with the C-terminal one-third part of nsp3 (nsp3_C_) resulted in the relocalization of these proteins from the ER into discrete foci mostly localizing to the perinuclear region of the cell [81]. Although nsp6 was not required for the observed relocalization, it was recruited to the perinuclear foci when co-expressed [82], in agreement with this protein interacting with nsp4 [81]. Ultrastructural analysis of cells co-expressing nsp4 and nsp3_C_ revealed that the membranes of the ER exhibited more curvature, although these membranes did not resemble the DMVs observed in CoV-infected cells [82], which may not be surprising as only the C-terminal part of nsp3 was used in these co-expressions with nsp4. Similar membrane rearrangements were not observed when the nsp3 from MHV was co-expressed with nsp4 from SARS-CoV (or vice versa), while (deletion) mutagenesis studies indicated essential roles for the large luminal loops of nsp3 and nsp4 in the relocalization of these proteins [82]. While co-immunoprecipitation and immunofluorescence assays indicate that nsp3 and nsp4 of MHV interact, this interaction could not be confirmed using the Venus protein-fragment complementation assay [81]. We hypothesize that the interactions between nsp3 and nsp4 mediate some kind of “zippering” of the lipid bilayers of the ER, which ultimately leads to the formation of the DMVs. In this model nsp3 and nsp4 interact via their luminal loops in such a way that their interaction prevents reconstitution of a functional Venus protein. 

Several other studies also suggest an important role for nsp4 in the generation of the replicative structures. Disruption of the nsp4 glycosylation sites present in the loop between the first and second hydrophobic region, leads to the formation of aberrant DMVs in which the inner and outer membranes are detached, while the number of CMs is increased [83]. In agreement herewith, although the fourth hydrophobic domain of nsp4 is dispensable, the other three transmembrane regions are required for CoV replication [84]. Furthermore, co-expression of the counterparts of the CoV nsp3 and nsp4 of the distantly-related equine arteritis virus (EAV), resulted in the rearrangement of host cell membranes into DMVs, albeit with a morphology [85] differing from that observed in EAV-infected cells [86]. Mutations of cysteine residues present in the luminal loop of EAV nsp3 (the EAV counterpart of CoV nsp4) resulted in altered morphologies of the DMVs, while the introduction of a *N*-glycosylation site in this loop also affected their morphology to some extent [87].

Like CoVs, other +RNA viruses also somehow induce membrane rearrangements that are required for their replication and transcription. For several of these viruses similar membrane rearrangements can be induced by the (co-)expression of nsps (for reviews see [1,2,3]). These nsps are either integral transmembrane proteins, examples being the NS4A proteins of Dengue virus (DENV) [88] and Kunjin virus [89] and the NS4B protein of hepatitis C virus (HCV) [90], or alternatively the proteins are only peripherally associated to the lipid bilayer, such as the 1a protein of brome mosaic virus (BMV) [91] and the 2C protein of poliovirus (PV) [92]. Strikingly, however, also for the integral membrane proteins the occurrence of hydrophobic/amphipathic regions that do not span the lipid bilayer but are peripherally associated with membranes, which has also been demonstrated for CoV nsp3 and nsp6 [42,44,45], appears to be a common feature [88,93,94,95]. Another similarity among the nsps of different +RNA viruses appears to be their ability to assemble into larger protein complexes. Such interactions have been observed not only between nsp3, nsp4 and nsp6 of CoVs, but also between the nsps of other +RNA viruses, including the BMV 1a protein [96,97] and the flavivirus NS4A and NS4B proteins [98,99].

How are the nsps of CoVs able to induce the observed membrane rearrangements? We speculate that the similarities between the membrane-associated nsps of different +RNA viruses relate to their common ability to remodel membranes. The induction of membrane curvature in lipid bilayers is critical when remodeling host cellular membranes. The membrane-associated viral proteins may act as multimeric scaffolds able to impose such curvature, for instance by acting as wedges by inserting their amphipatic helices partially into one side of the bilayer (reviewed in [100,101,102]). The viral proteins may function similar to host cellular proteins known to induce membrane bending via a scaffold mechanism, as exemplified by the COPI and COPII complexes, and/or via the insertion of amphipathic domains into the lipid bilayer, as has been proposed for the small GTPase Sar1 (reviewed in [100,102]).

## 5. Dynamics of CoV Replicative Structures and Associated Proteins

The last few decades have provided virologists with exciting new information regarding the functions and structures of individual nsps and the characterization and formation of the membranous structures induced by +RNA viruses. These insights were obtained by classical biochemical, immunofluorescent and ultrastructural approaches. Unfortunately, little information regarding the dynamics of the (viral) proteins present at the membranous replicative structures in living cells is known. Such studies are important as classical approaches only provide static views of cellular processes and do not necessarily reflect the dynamics underlying virus replication in living cells. To date, only few studies on the dynamics of the replicative structures of plus-strand RNA viruses and their associated proteins have been published [103,104,105,106,107,108,109].

### 5.1. Dynamics of the CoV Replicative Structures

By using a GFP-tagged version of nsp2, shown by immuno-electron microscopy (IEM) to be recruited to the DMVs and CMs, as a marker the dynamics of the CoV replicative structures were studied using live cell imaging [110]. These studies showed for the first time that the CoV replicative structures are moving through the cell. However, it appeared that the CoV replicative structures consist of two classes that demonstrate different mobilities: large structures lacking any displacement and smaller structures with relatively high saltatory mobility. The smaller replicative structures were hypothesized to correspond to individual DMVs, while the larger ones supposedly represent the DMV/CM assemblies that have been observed in ultrastructural studies of CoV-infected cells [4,62]. In SARS-CoV-infected cells the DMVs are confined to a reticulovesicular network [4]. We therefore speculate that the small structures have not yet been ‘captured’ into this network. However, correlative light-electron microscopy studies will be required to solve this issue. 

Live cell imaging studies of HCV- and Semliki Forest virus (SFV)-infected cells also reveal the presence of replicative structures that could be discriminated on the basis of their size and mobility. HCV induces the formation of a so-called membranous web, in which DMVs can be observed [90]. Large HCV structures, probably representing membranous webs, exhibited limited movement, whereas smaller ones were mobile and could travel long distances throughout the cytoplasm [108]. SFV-induced vacuoles are assembled at the plasma membrane after which they are transported to modified lysosomes [106,107]. SFV-infected cells also harbor large acidic immobile perinuclear vesicles and smaller acidic cytoplasmic vesicles that showed saltatory movements. In addition to these acidic vesicles, SFV-infected cells contain a class of non-acidic highly mobile vesicles that displayed multidirectional short-distance movements. Moreover, fusion of the neutral mobile structures with the acidic mobile structures resulted in the formation of the large acidic structures [107]. Such events of fusion of smaller replicative structures into larger ones have not been observed (yet) for the CoV and HCV replicative structures [108,110].

The calculated velocities of the saltatory movements of the smaller nsp2-positive structures in CoV-infected cells [110] correspond to those measured for microtubule-mediated transport [111]. The observed association of these structures with microtubules and the inhibition of trafficking in the presence of a microtubule network-disturbing drug confirmed the transport of the smaller structures on microtubular tracks [110]. A role of the cytoskeleton in the movement of replicative structures has also been described for HCV, SFV, and PV [105,107,108,109]. Disruption of a functional microtubular network inhibited the movement of the small HCV and nascent PV replicative structures [108,109] and the trafficking of SFV neutral vesicles to acidic organelles [107], concomitant with dispersal of these structures throughout the cytoplasm. Strikingly, inhibition of microtubule-dependent trafficking did not or only modestly affect the replication of these viruses [109,110,112]. Disruption of the actin network also did not affect CoV replication much, although the replicative structures again failed to accumulate in the perinuclear area [113]. Collectively, these results show that the cytoskeleton is required for the perinuclear accumulation of the replicative structure rather than for replication *per se*. Additional studies are required to clarify the role of the perinuclear targeting of the CoV replicative structures during infection.

### 5.2. Mobility of Replicative Structure-Associated Proteins

Up till now, only few studies have addressed the dynamics of individual viral proteins when present at the replicative structures. Recently, we analyzed the dynamic properties of three replication-associated proteins, *i.e*., the soluble nsp2 and N proteins and the integral membrane protein nsp4, and demonstrated that these proteins display different diffusional mobilities when present at the replicative structures.

Nsp2, when expressed *in trans* is recruited to the replicative structures [110]. After recruitment, nsp2 was shown to be immobilized by using fluorescent recovery after photobleaching (FRAP) methodology. In other words, nsp2 already associated with the replicative structures was not exchanged by nsp2 present at other locations in the cell. Similar results have been demonstrated for other +RNA virus replication-associated proteins, although the published literature on this subject is limited. Also in HCV-infected cells, NS5A-positive structures showed a static internal architecture when (part of) the NS5A fluorescent protein pool was bleached [108]. When expressed individually, NS5A is highly mobile [103], similar to nsp2. Apparently, in the context of a viral infection, when other viral proteins are present, MHV nsp2 and HCV NS5A are immobilized at the replicative structures presumably due to protein-RNA or protein-protein interactions. In agreement herewith, large-scale protein-protein interaction studies demonstrated that nsp2 of MHV and SARS-CoV is engaged in a multitude of interactions with itself, nsp3, nsp4, nsp6, nsp7, nsp8, nsp11, nsp15 and nsp16 [82,114,115,116]. These observations are remarkable in view of the dispensability of nsp2 during CoV infection *in vitro* [59]. Yet, nsp2 has to be somehow important *in vivo* as its coding sequence is maintained during evolution. 

In addition to its structural role in the coronavirion, *i.e.*, in packaging of the genomic RNA into the RNP complex, the structural N protein is also important in coronavirus replication and has been detected in the perinuclear region of infected cells colocalizing with markers for the replicative structures [62,67,117,118]. The interaction of the N protein with nsp3 is presumably important for the initial recruitment of N to the replicative structures [119], while N-N protein interactions were sufficient for recruitment of (mutant) N proteins in the presence of wild type N proteins [120]. In contrast to nsp2, the N protein is not immobilized at the CoV replicative structures but is associated with it rather dynamically [120]. This may not be surprising, as the multifunctional N protein is both involved in viral replication [121,122,123] and virion assembly [124]. As the CoV replicative structures and virion assembly sites appear to be spatially separated, the newly synthesized genomic viral RNA needs to be transported from the replication sites to the assembly sites. The N protein presumably facilitates this transport, consistent with its dynamic behavior.

When expressed *in trans* in infected cells, the nsp4-GFP fusion protein was detected at the ER and at the replicative structures [43,81]. By performing fluorescence loss in photobleaching (FLIP) experiments continuity was demonstrated between the membranes of the ER and the replicative structures that harbor nsp4 [81], in agreement with the model that the DMVs and CMs form an interconnected network that is continuous with the ER [4]. However, nsp4 displayed different diffusional mobilities at different subcellular locations [81]. It was more mobile in the ER than at the replicative structures. This reduced mobility may be (partly) due to its engagement in interactions with the other transmembrane-containing nsps as well as with itself [81]. Also the mobility of the HCV NS4B protein, which is an integral transmembrane protein as well, depends on its intracellular location. When expressed in the absence of other viral proteins, this protein is present at the ER and at so-called membrane-associated foci (MAFs) that are induced upon expression of this protein [104]. FRAP analysis showed that NS4B present at the MAFs had a reduced mobility compared to NS4B at the ER, which was suggested to result from NS4B being engaged in different interactions when present on MAFs or the ER [104].

## 6. CoV Replicative Structures and RNA Synthesis

Currently, one of the most enigmatic issues regarding CoV replication is the precise localization of the sites of active viral RNA synthesis. Although CoV RNA synthesis appears to be protected by membranes [125] it is still unclear whether synthesis of nascent viral RNA occurs at sites of dsRNA accumulation, as pores connecting the interior of the coronavirus DMVs with the cytoplasm have not been detected [4], while the nsps localize to both DMVs and CMs [4,5,19,43,59,62,63,68,69,70,71]. To identify sites of nascent viral RNA synthesis, one has to define what actually constitutes the active viral replication complexes. Although dsRNA molecules function as intermediates of replication and transcription, their presence at certain sites *per se* does not imply (all) these structures to be actively involved in RNA synthesis. Likewise, the location of viral enzymes that are required for RNA synthesis does not need to correlate with active RTCs. Moreover, newly synthesized RNAs are not necessarily located at their site of synthesis as they may diffuse or be transported away to other subcellular locations. In view of these considerations, sites active in RNA synthesis are expected to contain at least three components: the RdRp, dsRNA intermediates active in replication/transcription and nascent viral RNA.

Newly synthesized viral RNAs, visualized by 5-bromouridine 5’-triphosphate (BrUTP) labeling, were shown to colocalize with antibodies recognizing either nsp5 or the C-terminal part of pp1a [71], and were observed in close proximity to the DMVs by immunoelectron microscopy in MHV-infected cells [47,71]. Recently, a new method was used to detect and visualize newly synthesized coronaviral RNA by incorporation of an alkyne-modified uridine analog, 5-ethynyl uridine (EU), onto which an azide-derivatized fluophore was coupled via a copper (I)-catalyzed cycloaddition reaction (click chemistry). With this method, it was shown that throughout MHV infection foci of nascent RNAs could be detected, which colocalize with the RdRp-containing nsp12, indicating that they correspond with sites of active coronaviral RNA synthesis. The relationship between nascent RNA and dsRNA is, however, less clear. While early in infection nascent RNAs colocalize at or adjacent to patches of dsRNA dots, presumably corresponding to DMVs, this correlation is much less apparent at later times when the dsRNA dots are spread throughout the cell. Many dsRNA dots are apparently not transcriptionally active as no EU labeling was associated with them, while many foci of EU labeling did not appear to colocalize with the dsRNA dots [113].

Different models can be put forward to explain these observations. In one model, DMVs function as the sites of active RNA synthesis. At the later times in infection, many DMVs are no longer active, while the ones that are active may contain only little dsRNA, resulting in less apparent colocalization of dsRNA and EU labeling. In another model, DMVs are non-functional end-stage products. They are not actively involved in RNA synthesis, but rather harbor dsRNAs that are not (longer) functioning as intermediates in RNA synthesis. In this model, which is in agreement with the presumed absence of pores in these structures [4], the CMs would be the only plausible alternative for the sites of active RNA synthesis. In yet another model, DMVs may be the initial sites of active RNA synthesis, particularly early in infection, while at later times the membranes become sealed, connections are lost and RNA synthesis shifts to the CM assemblies. Clearly, ultrastructural studies will be required, ideally (co)localizing nascent RNAs as well as the RdRp, to definitely determine the precise localization of CoV RNA synthesis.

For most other +RNA viruses the identification of the sites of active RNA synthesis appears less complicated. Nascent RNAs, as well as nsps, have been shown to label the spherules that are observed in BMV- [126], SFV- [106] or flock house virus (FHV)- [127] infected cells at the modified ER, lysosomes and mitochondria, respectively, indicating that these structures correspond with the sites of active RNA synthesis. Nascent RNAs were previously also shown to colocalize with dsRNA in Kunjin flavivirus-infected cells [128]. Electron tomography of the membrane rearrangements observed in flavirus-infected cells revealed that the inner content of the DMVs, which contains nsps and dsRNA, is connected to the cytoplasm via a pore [129,130], indicating that these DMVs are actually spherule-like invaginations (once) active in RNA synthesis.

## 7. Future Directions

CoV replicative structures/RTCs are macromolecular assemblies, the components of which are engaged in a plethora of protein-protein, protein-RNA, and protein-lipid interactions [114,115,116,131]. Currently, the exact composition of the replicative structures/RTCs is not known, let alone the full arsenal of interactions occurring within these structures. Moreover, the replicative structures are likely to be subject to some form of maturation [14,19,20,113], as their composition appears to change during the course of infection as determined by biochemical and immunofluorescence analyses. Thus, it will be of interest to confirm, extend and refine the previously published protein-protein interactome studies that have been published for SARS-CoV [114,115,116], for example by investigating protein-protein interactions of other CoVs using novel (large-scale) screening approaches. Likewise, it will be of interest to get more insight into the involvement of host proteins in the formation of these structures, for example by screening for host proteins that interact with the CoV nsps or by elucidating the protein content of purified replicative structures -preferably in time- using mass spectrometry.

One of the difficulties associated with these studies is the complexity of discriminating whether host proteins are directly involved in the formation of the replicative structures themselves or in RNA synthesis *per se*, as inhibition of either process will result in reduced RNA synthesis, protein expression and DMV formation. Therefore, assays are needed in which the formation of replicative structures can be studied independent of viral replication. Such assays may be provided by co-expression of viral proteins [81,85] that induce the rearrangement of cellular membranes. Recently, by using such a replication-independent assay, reticulons, which form a family of ER membrane-shaping proteins, were implicated in the formation of the BMV-induced, ER-derived spherules that are associated with viral RNA synthesis [132]. Reticulons are involved in the induction of ER membrane curvature [133,134,135] and seem to be required by BMV for determining the size of the spherules and for stabilizing the spherule necks [132]. It will be of interest to study the putative role of these proteins in CoV replication and in reshaping of the ER membranes by CoV nsps. Other proteins playing a role in shaping and remodeling of the ER are the atlastins and CLIMP-63 [133,136]. Also these proteins are putative candidates involved in CoV-induced membrane remodeling and would deserve to be investigated.

In addition to the role of virus and host proteins in formation of the replicative structures, lipids may also be important in this process. Dynamic alteration of the lipid composition can induce curvature of membranes to some extent [100,102,137,138], which is, however, unlikely to be sufficient to generate the organelle-like structures observed in +RNA virus-infected cells. It is more likely that lipids, together with lipid-modifying enzymes, contribute to the formation of the replicative structures by providing a suitable microenvironment to which the viral (and cellular) membrane shaping proteins are recruited. Even more, +RNA viruses may specifically hijack lipid-modifying enzymes for their own advantage [139,140]. This is underscored by several studies, which show that inhibition of lipid synthesis by using either drugs or small interfering RNAs dramatically affects the replication of +RNA viruses [139,141,142,143,144]. It will be of interest to study how and to what extent CoV infection affects the lipid homeostasis in infected cells, for example by analyzing the lipid repertoire of infected cells by lipidomics techniques.

Finally, as all processes occurring in living cells are inherently dynamic in nature, it is also desirable to get more insight into the biogenesis and functioning of the replicative structures using various live cell imaging approaches. For example, photoactivatable fluorescent proteins can be used to investigate the formation of the replicative structures in real-time by ‘optical pulse-labeling’ in living cells, while the behavior of individual nsps associated with the RTCs can be studied by selectively ‘switching-on’ (sub)populations of proteins [145,146]. Furthermore, tagging of viral RNA by genetic incorporation of specific RNA sequences that bind fluorescently-tagged RNA-binding proteins [147,148], hybridization of fluorescent ‘molecular beacons’ to the viral RNAs [149], or combining metabolic labeling of viral RNAs with Cu-independent click chemistry [150], will allow visualization and tracking of these ribonucleic acid species in living cells. Concomitant live cell imaging of viral RNA and the N proteins may be used to investigate the transport of nascent RNA from the replication to the assembly sites. In addition, super resolution microscopy techniques, like photoactivated localization microscopy (PALM), stimulated emission depletion microscsopy (STED) and stochastic optical reconstruction microscopy (STORM) [151,152,153] can all be applied to investigate the CoV RTCs and the membranous replicative structures at the ultrastructural level using fluorescently-tagged proteins, while EU-labeling of viral RNA in combination with correlative light-electron microscopy may provide the resolution to indisputably pinpoint the exact location of viral RNA synthesis [154].
